# Sedentary Lifestyle Is a Modifiable Risk Factor for Cognitive Impairment in Patients on Dialysis and after Kidney Transplantation

**DOI:** 10.3390/jcm13206083

**Published:** 2024-10-12

**Authors:** Aleksandra Golenia, Piotr Olejnik, Oliwia Maciejewska, Ewa Wojtaszek, Paweł Żebrowski, Jolanta Małyszko

**Affiliations:** 1 Department of Neurology, Medical University of Warsaw, 02-091 Warsaw, Poland; piotrek.olejnik2001@gmail.com (P.O.); maciejewska@autograf.pl (O.M.); 2 Department of Nephrology, Dialysis and Internal Medicine, Medical University of Warsaw, 02-091 Warsaw, Poland; ewa.wojtaszek@wum.edu.pl (E.W.); pawel.zebrowski@wum.edu.pl (P.Ż.); jolmal@poczta.onet.pl (J.M.)

**Keywords:** cognitive impairment, end-stage kidney disease, renal replacement therapy, modifiable risk factors, dementia, sedentary lifestyle, educational attainment

## Abstract

**Background:** Chronic kidney disease (CKD) is a risk factor for cognitive impairment (CI), and this risk is the highest in patients with end-stage kidney disease (ESKD). As a multifactorial disease, CI may be influenced by several potentially modifiable lifestyle and behavioral factors that may reduce or increase the risk of dementia. The aim of this study was to evaluate the associations between the known modifiable risk factors for dementia and the risk of CI in patients with ESKD treated with renal replacement therapy. The Charlson Comorbidity Index and the risk of CI in patients with ESKD were also assessed. **Methods:** In this cross-sectional study, 225 consecutive patients with ESKD treated with different modalities of renal replacement therapy were assessed for cognitive decline using the Addenbrooke’s Cognitive Examination (ACE III) test. Information was also collected on modifiable risk factors for dementia, medical history and demographics. **Results:** This study included 117 patients after kidney transplantation (KT) and 108 patients with ESKD undergoing peritoneal dialysis and hemodialysis. The prevalence of modifiable risk factors for dementia differed between the groups; KT patients were more likely to be physically active, residing in cities with populations of less than 500,000 inhabitants, and were less likely to suffer from depression. Furthermore, the KT group had a lower Charlson Comorbidity Index score, indicating less severe comorbidities, and a lower risk of CI (3.6 ± 1.67 vs. 5.43 ± 2.37; *p* = 0.001). In both the KT and dialysis groups, patients with CI were more likely to have a sedentary lifestyle (45% vs. 9%, *p =* 0.001 and 88% vs. 48%, *p =* 0.001, respectively), whereas lower educational attainment and depression had a significant negative impact on ACE III test results, but only in KT patients. Finally, cognitive function in dialysis patients was negatively affected by social isolation and living in urban areas. **Conclusions:** Modifiable risk factors for dementia, particularly a sedentary lifestyle, are associated with a higher risk of CI in patients treated with different renal replacement therapy modalities. As CI is an irreversible condition, it is important to identify lifestyle-related factors that may lead to dementia in order to improve or maintain cognitive function in patients with ESKD.

## 1. Introduction

Chronic kidney disease (CKD) is a risk factor for cognitive decline [1]. The risk of cognitive impairment (CI) increases with the stage of CKD and is highest in patients with end-stage kidney disease (ESKD) requiring renal replacement therapy [2,3]. As we have shown in our previous reports, the prevalence of CI ranges from 30% in kidney transplant recipients and 33% in peritoneal dialysis patients to 58% in hemodialysis patients [4,5,6]. The possible mechanisms contributing to CI in CKD are not fully understood. On the one hand, there are widely acknowledged traditional risk factors for cardiovascular disease, as well as factors associated with kidney disease, such as chronic inflammation, elevated concentrations of uremic toxins, malnutrition, and alterations in cerebral blood flow, that may lead to CI in patients with CKD [7]. Numerous studies have shown that CKD is closely associated with low-grade systemic inflammation and oxidative stress, which then contribute to various complications [8], including CI. The Chronic Renal Insufficiency Cohort (CRIC) study, conducted on a large cohort of 757 CKD patients, found an association between elevated levels of high-sensitivity C-reactive protein, fibrinogen, and interleukin-1β (IL-1β) and an increased risk of impaired attention [9]. Nevertheless, the same study showed that higher levels of tumor necrosis factor-α (TNF-α), another pro-inflammatory cytokine, were linked to a lower risk of executive dysfunction [9]. Furthermore, preclinical studies have consistently supported the concept that CI is an immune-mediated process in the course of CKD. Zimmermann et al. demonstrated the pro-inflammatory activation of microglia in an animal model of CKD that may be responsible for the development of CI [10]. In addition, malnutrition is a common comorbid condition of ESKD. According to a study by Rotondi et al., malnutrition, as assessed by the malnutrition scale, has been identified as a risk factor for CI in hemodialysis (HD) patients [11].

On the other hand, as a multifactorial disease, CI may be influenced by several potentially modifiable lifestyle and behavioral risk factors that may reduce or increase the risk of dementia [12,13].

There are 12 known modifiable risk factors for dementia, including lower educational attainment, hearing loss, traumatic brain injury, hypertension, excessive alcohol consumption, obesity, smoking, depression, social isolation, physical inactivity, air pollution, and diabetes [1]. These modifiable risk factors have been shown to be associated with 40% of all cases of dementia [14]. Given that dementia is irreversible, it is important to identify the ways of preventing the onset and slowing the course of the disease. A large study, the Finnish Geriatric Intervention Study to Prevent Cognitive Impairment and Disability (FINGER), found that multi-domain lifestyle interventions can increase cognitive reserve and reduce inflammation and vascular or oxidative damage in the brain in elderly people at increased risk of dementia and without obvious cognitive problems [15,16].

The link between CI in patients with ESKD and modifiable risk factors for dementia has not been sufficiently studied. Therefore, the aim of the present study was to evaluate the associations between known modifiable risk factors for dementia and the risk of CI in patients with ESKD treated with renal replacement therapy. The Charlson Comorbidity Index (CCI) and the risk of CI in patients with ESKD were also assessed.

## 2. Materials and Methods

This cross-sectional study included patients with ESKD treated with different modalities of renal replacement therapy, such as peritoneal dialysis (PD) and hemodialysis, and patients after kidney transplantation (KT). Patients who consented to this study were recruited between 1 August 2022 and 31 May 2024. Patients were enrolled in this study if they had ESKD, were on PD, ambulatory HD, and after KT, were 18 years of age or older, were Polish speakers, and previously had a consultation with the treatment team. To reduce the number of false positives, only clinically stable patients without infectious diseases in the last 8 weeks, decompensated heart, liver failure, psychiatric or neurodegenerative disorders, or delirium were enrolled in this study. Exclusion criteria also included language barriers, physical disabilities such as visual impairment, limb paresis, and previous severe cognitive problems, as well as the use of hypnotics (including benzodiazepines and z-drugs)

Demographic data (age and gender) and medical history (duration of dialysis or time from KT to cognitive assessment and comorbidities) were obtained from hospital medical records. In addition, information was obtained from all study participants on other modifiable risk factors for dementia, such as years of education; hearing impairment (yes/no); physical activity; living alone or with others; smoking (never/current); excessive alcohol consumption (yes/no); traumatic brain injury requiring hospitalization, or brain contusion or concussion (yes/no); full-time employment (yes/no); and living in urban areas—cities with more than 500,000 inhabitants (yes/no).

According to World Health Organization guidelines and recommendations, regular physical activity was defined as at least 150 min of moderate-intensity aerobic activity or 75 min of vigorous-intensity aerobic activity per week [17]. Responses were dichotomous, with regular physical activity once a week scored as zero and no physical activity scored as one.

We used the Charlson Comorbidity Index (CCI) which is the most commonly used tool to measure concomitant conditions and consists of 19 items corresponding to different comorbidities [18]. CCI not only accounts for the number of concomitant diseases but also considers the severity of each condition by assigning a weighted score to reflect the burden of disease. For example, a history of myocardial infarction or cerebrovascular disease is assigned a weight of 1, while conditions such as localized tumors, lymphoma or leukemia are weighted at 2. The most severe conditions, such as metastatic neoplasms or acquired immunodeficiency syndrome, receive a weight of as much as 6. The maximum score a patient could achieve was 37 points. The higher the score, the greater the risk of mortality and poor outcome due to the presence of more severe comorbid conditions [18].

### 2.1. Cognitive Function Assessment

Cognitive impairment was assessed using the Addenbrooke’s Cognitive Examination III (ACE III) test, a detailed description of which has been described elsewhere [4]. In brief, this test assesses five major cognitive domains, including attention, verbal fluency, memory, language, and visuospatial abilities, with a score ranging from 0 to 100 points, and takes 15 to 20 min to complete. KT and PD patients were assessed in the morning (between 8:00 and 12:00), in a separate room, in silence, during a scheduled visit to the transplant clinic. HD patients were assessed during the first hours of the HD session and at approximately the same time. All assessments were performed by one of two investigators. Cutoff points of ≤88 were used for CI estimation, including both mild CI and suspected dementia [19].

The local ethics committee approved the study protocol (approval number KB/81/2022). This study followed the World Medical Association Declaration of Helsinki. Written informed consent was obtained from all participants.

### 2.2. Statistical Analysis

The analysis was performed using IBM SPSS Statistics 29.0 software. The Shapiro–Wilk test was used to analyze the data. A non-parametric test, the Mann–Whitney U test, was used to test for differences in various parameters due to non-normal distribution. A chi-squared test of independence was used to compare the groups in question. Hierarchical regression analysis was performed to determine the factors influencing the ACE III test results. The dependent variable showed a normal distribution. A two-step procedure was used. In the initial regression model, modifiable risk factors for dementia were introduced as independent variables. In the subsequent model, CCI, age, and time from KT to assessment or dialysis duration were included as additional independent variables. A significance level of *p* < 0.05 was used.

## 3. Results

### 3.1. Baseline Characteristics of the Study Population

This study included 117 consecutive patients after KT and 108 consecutive patients with ESKD undergoing PD or HD. The demographic and clinical characteristics as well as the comparison of risk factors for dementia of the study groups are summarized in Table 1. The age distribution differed between the groups, with the KT group being younger. Similarly, the KT group had a lower CCI score, indicating less severe comorbidities, and a lower risk of CI (3.6 ± 1.67 vs. 5.43 ± 2.37). The mean dialysis duration was 28 months, and the mean time from KT to cognitive assessment was 10 months. Finally, the prevalence of modifiable risk factors differed between the groups (Table 1). Physical inactivity and depression were more prevalent in patients undergoing PD and HD than in the KT group. Furthermore, participants undergoing PD and HD predominantly lived in urban areas with populations of over 500,000 residents, while KT patients lived in cities with populations of less than 500,000 residents or in rural areas.

### 3.2. Modifiable Risk Factors for Dementia and ACE III Test Results

**Figure 1:**
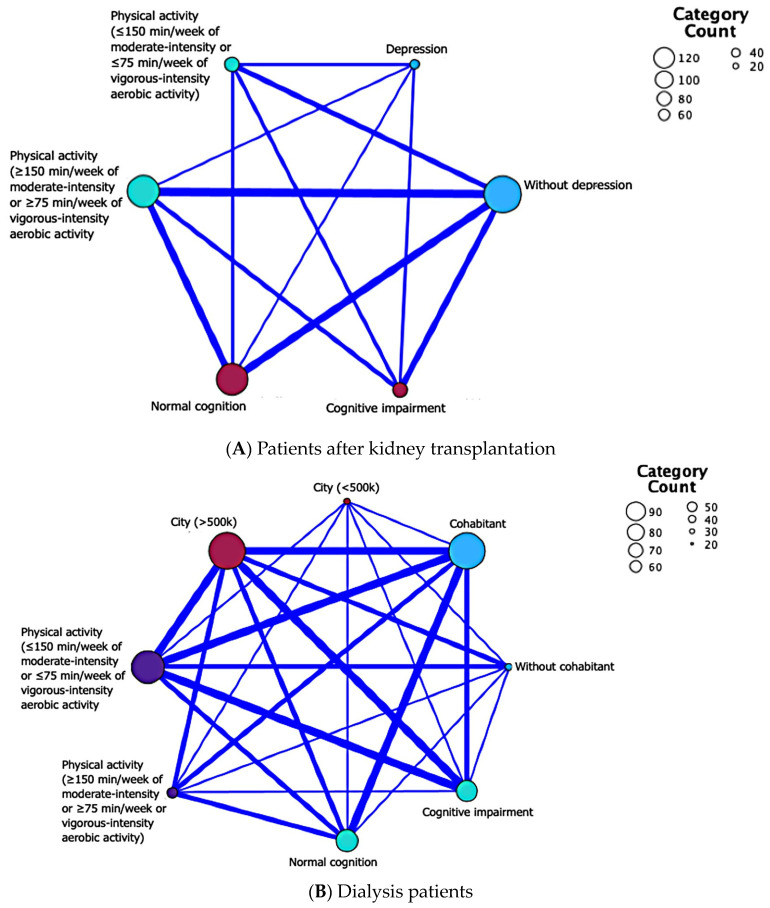
Relationship map summarizing the inter-relationships between variables in each subpopulation—(**A**) kidney transplant recipients and (**B**) dialysis patients. Only variables statistically significantly different between normal cognition and CI are presented. The thicker the line connecting two variables is, the more the patients manifested both characteristics simultaneously. For instance, in part 1 a, the majority of patients presenting normal cognition had at least 150 min of moderate-intensity or 75 min of vigorous-intensity aerobic activity per week and did not have symptoms of depression in the screening test. Additionally, in part 1 b, most patients with cognitive decline lived in a city with a population of more than 500,000 residents and had less than least 150 min of moderate-intensity or 75 min of vigorous-intensity aerobic activity per week.

Exposure to modifiable dementia risk factors in the study groups and their impact on cognitive function are presented in Table 2. In both the KT and the dialysis group, patients with CI were more likely to have a sedentary lifestyle (45% vs. 9%, *p =* 0.001 and 88% vs. 48%, *p =* 0.001, respectively; Figure 1). Furthermore, lower educational attainment and depression had a significant negative impact on the ACE III test results but only in KT patients (Table 2 and Figure 1). In addition, in the dialysis population, the risk factors for dementia that negatively affected cognition were social isolation and living in urban areas. Patients with CI were more likely to live alone and to live in cities with more than 500,000 inhabitants (Table 2 and Figure 1). Finally, dialysis patients with CI had a higher CCI score than dialysis patients without CI (6.38 ± 2.4 vs. 4.51 ± 1.96; *p* = 0.001; Table 2), but this correlation was not observed in the KT group (4.03 ± 1.74 vs. 3.44 ± 1.63; *p* = 0.06; Table 2).

### 3.3. Predictors of Cognitive Impairment in KT and Dialysis Patients

CI risk factors were divided into two blocks of variables and then analyzed individually using hierarchical multiple regression analysis. For the first model, we entered the modifiable risk for dementia. For the second model, we entered age, CCI, and time from kidney transplantation to cognitive evaluation or dialysis duration as additional independent variables. Table 3 shows the model summary of the R-square and R-square change associated with each step in the hierarchical multiple regression.

Overall, the first model, with an R-square of 0.23 in post-KT patients and 0.27 in dialysis patients, was statistically significant in both groups of patients and suggested that modifiable risk factors for dementia accounted for 23% and 27% of the variability in ACE III test results, respectively (Table 3). The second model, which included age, CCI, and time from kidney transplantation to cognitive assessment or dialysis duration showed a significant improvement over the first model but only in dialysis patients *F* (3, 91) = 13.78, *p* < 0.001, *R^2^* = 0.50. Overall, when age, CCI, and duration of dialysis were included in the model, these variables accounted for 50% of the variability in the ACE III test results in the dialysis group. There was no noticeable improvement in the KT patients *F* (3, 101) = 1.02, *p=* 0.38, *R^2^* = 0.25. In addition, depression (β= −0.3) in KT patients and age (β = −0.43) in dialysis patients negatively affected cognitive function, while physical activity (β = 0.41) had a positive effect on cognition, but only in dialysis patients.

## 4. Discussion

In the present study, we investigated the exposure to known modifiable risk factors for dementia and their associations with CI in kidney transplant recipients as well as in patients undergoing PD and HD. A sedentary lifestyle was associated with a higher risk of CI in both study groups.

Further, dialysis patients with CI were more likely to live alone and in urban areas. Moreover, depression and lower educational attainment were associated with cognitive decline but only in KT patients. In addition, KT patients had a lower CCI score, indicating less severe comorbidities, and a lower risk of CI compared to the dialysis group. Finally, dialysis patients with CI had a higher CCI score than dialysis patients without CI, but this correlation was not observed in the KT group. The results of the hierarchical multiple regression analysis showed that cognitive function in dialysis patients depended not only on modifiable risk factors for dementia but also on age, while this effect was not observed in KT patients.

Our results are broadly consistent with research describing the beneficial effects of physical activity on cognitive function [20,21]. As shown in previously published studies, physical activity has a positive effect on improving cognitive function in the general population [20] as well as in people with chronic diseases, regardless of the type of disease [22]. Furthermore, patients with advanced stage CKD and CI had lower levels of physical activity compared to patients with CKD and normal cognitive performance [23]. Additionally, a recent review suggests that physical activity may improve or at least not worsen cognitive function in patients with ESKD treated with HD [24].

The association between physical activity and CI can be explained by several mechanisms. First, aerobic exercise improves cognitive function at three levels: the systemic level, the cellular level (synaptic plasticity, neurogenesis, and angiogenesis), and the molecular level (neurotrophins and growth factors) is associated with changes in brain volume, cerebral blood flow, growth factor availability, and signaling cascades [21]. Further, individuals in good physical condition are able to tolerate a higher neuropathologic load before the clinical features of CI become apparent [25]. Finally, physical activity may reduce inflammation and thus improve brain plasticity, leading to better cognitive performance [24].

Data on exercise as an intervention to preserve cognitive function in dialyzed patients are limited. The EXCITE (Exercise Introduction to Dialysis Performance Improvement) study demonstrated that a home-based, personalized exercise intervention improved self-rated cognitive function [26]. In addition, in dialysis patients over 65 years of age, exercise preserved self-reported cognitive function [27]. Other small trials reported that either the exercise or cognitive training preserved cognitive function in dialyzed population [28,29]. Chu et al. summarized data on effective interventions for preserving cognitive function and prevent cognitive decline in dialyzed patients and/or kidney transplant recipients [30]. They stressed that cognitive prerehabilitation, with cognitive and/or exercise training, could serve as a desired interventions for potential kidney transplant recipients to prevent dementia but also to reduce delirium risk and long-term cognitive decline after transplantation [30].

Our study also showed that patients with higher educational attainment among KT recipients had better cognitive screening test results, indirectly suggesting that better education may play a protective role against cognitive decline. It is well known that patients with ESKD are more likely to develop brain abnormalities on magnetic resonance imaging, such as more severe white matter hyperintensities or brain atrophy, and consequently, cognitive problems may appear at a younger age [3]. There is evidence that individuals with higher education maintain cognitive function longer, even in the presence of white matter damage [31]. Further, cognitive performance is largely dependent on cognitive reserve, with years of education being the most commonly used proxy for cognitive reserve [32]. Individuals with higher levels of cognitive reserve can tolerate more brain pathology before cognitive decline occurs [33]. In addition, cognitive reserve can be increased by an enriched environment that offers enhanced sensory, cognitive, and motor stimulation, such as cohabitation [34]. Single, separated, or widowed individuals had 3 times the risk of CI and 7.67 times the risk of Alzheimer’s disease compared to their married or cohabiting counterparts [35].

In the present study, depression had a significant impact on cognitive decline but only in KT patients. One explanation may be that KT recipients were younger than patients undergoing PD and HD and may have greater concerns about social and occupational limitations, expected treatment duration, and treatment-related complications.

Depression is one of the most common mental disorders in patients with ESKD [36], with a prevalence of up to 75% [37] and higher rates in patients undergoing PD and HD compared to kidney transplant recipients [4,6,38]. A recent study found that depression is independently associated with an increased risk of cognitive decline in older adults [39], affecting memory, attention, and psychomotor speed in particular [40]. Furthermore, depression in patients undergoing renal replacement therapy was associated with an increased likelihood of adverse outcomes, such as higher rates of hospitalization or dialysis withdrawal, higher risk of peritonitis in PD patients, cardiovascular disease, and mortality [38].

Air pollution has recently been recognized as a modifiable risk factor for dementia. Specific pollutants, such as particulate matter 2.5 (PM_2.5_), nitrogen dioxide (NO_2_), and carbon monoxide (CO), have been identified as having a detrimental impact on cognitive function [14]. A possible mechanism is that particulate matter may affect the function of the blood–brain barrier, allowing the pro-inflammatory cytokines (e.g., IL-1β, TNF-α) to infiltrate. Such an infiltration could subsequently trigger neurodegenerative processes [41]. According to a systematic review by Peters et al., increased exposure to these air pollutants correlates with an elevated risk of cognitive decline [42]. A large cohort study by Chen et al., involving over 2 million Ontario residents, found a significant association between exposure to PM_2.5_ and NO_2_, and the incidence of CI [43]. These scientific findings are consistent with the results of our study, which demonstrated that dialysis patients residing in cities with populations of more than 500,000 inhabitants had a significantly higher incidence of CI. However, no significant statistical differences were observed in the KT group. This may be due to the fact that most dialysis patients reside in Warsaw, while KT patients are dispersed across various Polish cities. Although Warsaw is not the most polluted city in Poland, it is plausible that the composition of its pollution differs from that of other smaller Polish cities and villages.

The present study also showed that a higher CCI score may indicate a higher risk of CI in the dialysis population but not in the KT group. Further, dialysis patients have a high prevalence of comorbidities linked to an increased risk of CI [3]. Diabetes mellitus, hypertension, coronary artery disease, and cerebrovascular disease are important risk factors with a proven adverse effect on cognitive function [3]. Finally, dialysis patients are at an increased risk of CI due to a higher burden of traditional cardiovascular risk factors and diabetes mellitus combined with kidney-specific and dementia-related modifiable risk factors [3].

## 5. Limitations

There are several limitations to this study. First, its cross-sectional design makes it impossible for the results to be used to prove causality. Second, there was no healthy control group included in the study. Third, information on obesity, one of the modifiable risk factors for dementia, was not included in the analysis. Additionally, we used a proxy variable for air pollution instead of actual data on air pollution concentrations. However, obtaining accurate measurements of pollution was not possible due to the wide geographic distribution of patients in Poland and the unavailability of such data in many regions. Moreover, some risk factors were self-reported by patients, which may introduce recall bias and affect the accuracy of the data. Finally, some dialysis-specific variables, such as dialysis adequacy and modality, were not included in the analysis due to the small size of the dialysis subpopulation, which would limit the representativeness of the results.

The strength of this study lies in the fact that it assessed nearly all known modifiable dementia risk factors in combination with comorbidities, making the study more reliable.

In conclusion, we have shown that modifiable dementia risk factors, particularly a sedentary lifestyle, are associated with a higher risk of CI in patients treated with different renal replacement therapy modalities. As CI is an irreversible condition, it is important to identify lifestyle-related factors that may lead to cognitive decline in order to improve or maintain cognitive function in patients with ESKD. Therefore, potential interventions such as home-based and intradialytic exercise training and/or cognitive training are worth considering in efforts to preserve cognitive function in this vulnerable population.

## Figures and Tables

**Table 1:** Characteristics of the study group.

	KT (*N* = 117)	PD and HD (*N* = 108)	*p*-Value *
Age (years) *mean ± SD* [Q1; Q3]	50.9 ± 12.24 [44.5; 60.5]	61.59 ± 16.33] [51; 72]	0.001 ^a,^*
CCI *mean ± SD* [Q1; Q3]	3.60 ± 1,67 [2; 4]	5.43 ± 2.37 [4; 7.8]	0.001 ^a,^*
Modifiable risk factors for dementia			
Hypertension (*N*)	107	94	0.207 ^b^
Years of education *mean* ± *SD*	14.5 ± 3.38	14 ± 3,36	0.500 ^a^
[Q1; Q3]	[12; 17]	[12; 17]	
Diabetes mellitus (*N*)	29	35	0.284 ^b^
Living place (*N*) city with over 500,000 residents city with less than 500,000 residents			0.001 ^b,^*
	42	85	
	75	23	
Occupation (*N*)	109	99	0.671 ^b^
Cohabitant (*N*)	96	84	0.423 ^b^
Excessive alcohol consumption (*N*)	4	7	0.287 ^b^
Hearing impairment (*N*)	7	12	0.167 ^b^
Smoking (*N*)	10	21	0.062 ^b^
Physical activity (*N*) ≥150 min/week of moderate-intensity aerobic activity or ≥75 min/week of vigorous-intensity aerobic activity	87	31	0.001 ^b,^*
Traumatic brain injury (*N*)	12	14	0.526 ^b^
Depression (*N*)	13	23	0.037 ^b,^*

KT: kidney transplantation; PD: peritoneal dialysis; HD: hemodialysis; CCI: Charlson Comorbidity Index; N: number of patients; SD: standard deviation; Q1: quartile 25’; Q3: quartile 75’. * *p* < 0.05. ^a^ U Mann–Whitney test. ^b^ Chi-Squared. χ^2^.

**Table 2:** Influence of modifiable risk factors for dementia and CCI on cognitive function.

	KT (*N* = 117)			PD and HD (*N* = 108)		
	NonCI (*N* = 86)	CI (*N* = 31)	*p*-Value *	NonCI (*N* = 55)	CI (*N* = 53)	*p*-Value *
Age (years) *mean* ± *SD* [Q1; Q3]	50.07 ± 11.99 [43.75; 59]	53.19 ± 12.84 [46; 64]	0.18 ^a^	55.38 ± 15.97 [43; 69]	68.04 ± 14.16 [62; 77]	0.001 ^a,^*
Time from KT to cognitive assessment (months) *mean* ± *SD* [Q1;Q3]	10.75 ± 30.17 [1.13; 12.9]	9.67 ± 11.61 [1.17; 18.43]	0.39 ^a^	-	-	-
Duration of dialysis (months) *mean* ± *SD* [Q1; Q3]	-	-	-	17.4 ± 18.62 [5; 20]	39.71 ± 54 [7; 59]	0.17 ^a^
CCI mean ± *SD* *[Q1; Q3]*	3.44 ± 1.62 [2; 4]	4.03 ± 1.74 [3; 5]	0.06 ^a^	4.51 ± 1.96 [3; 6]	6.38 ± 2.40 [4.5; 8]	0.001 ^a,^*
Modifiable risk factors for dementia						
Hypertension (*N*)	78	29	0.626 ^b^	50	44	0.22 ^b^
Years of education mean ± SD [Q1; Q3]	14.86 ± 3.23 [12; 17]	13.52 ± 3.6 [11; 16]	0.027 ^a,^*	13.49 ± 3.02 [12; 16]	14.55 ± 3,63 [12; 17.5]	0.12 ^a^
Diabetes mellitus (*N*)	22	7	0.74 ^b^	17	18	0.74 ^b^
Living place (*N*) city with over 500,000 residents city with less than 500,000 residents	32 54	10 21	0.62 ^b^	38 7	47 6	0.013 ^b,^*
Occupation (*N*)	81	28	0.475 ^b^	50	49	0.772 ^b^
Cohabitant (*N*)	73	23	0.18 ^b^	48	36	0.016 ^b,^*
Excessive alcohol consumption (*N*)	3	1	0.95 ^b^	3	4	0.66 ^b^
Hearing impairment (*N*)	6	1	0.45 ^b^	5	7	0.49 ^b^
Smoking (*N*)	8	2	0.63 ^b^	9	12	0.41 ^b^
Physical activity (*N*) ≥150 min/week of moderate-intensity aerobic activity or ≥75 min/week of vigorous-intensity aerobic activity	71	16	0.001 ^b,^*	26	5	0.001 ^b,^*
Traumatic brain injury (*N*)	6	6	0.05 ^b^	4	10	0.07 ^b^
Depression (*N*)	5	8	0.002 ^b,^*	10	13	0.42 ^b^

NonCI: normal cognition; CI: cognitive impairment; KT: kidney transplantation; PD: peritoneal dialysis; HD: hemodialysis; CCI: Charlson Comorbidity Index; N: number of patients; SD: standard deviation; Q1: quartile 25’; Q3: quartile 75’; * *p* < 0.05. ^a^ U Mann–Whitney test. ^b^ Chi-Squared. χ^2^.

**Table 3:** Hierarchical regression analysis of predictors of ACE-III test results.

	KT (*N* = 117)				PD and HD (*N* = 108)			
	Model 1		Model 2		Model 1		Model 2	
Modifiable Risk Factors for Dementia								
	*β*	*p*	*β*	*p*	*β*	*p*	*β*	*p*
Hypertension	−0.107	0.26	−0.096	0.31	−0.073	0.46	−0.009	0.91
Years of education	0.034	0.71	0.028	0.77	−0.083	0.36	−0.129	0.09
Diabetes mellitus	−0.047	0.61	0.040	0.70	0.015	0.87	0.111	0.23
Living place	0.180	0.06	0.189	0.04	−0.105	0.29	0.019	0.83
Occupation	0.058	0.52	0.061	0.51	0.074	0.45	0.190	0.06
Cohabitant	0.068	0.45	0.054	0.55	0.085	0.36	−0.027	0.74
Excessive alcohol Consumption	−0.040	0.67	−0.053	0.57	−0.052	0.58	−0.023	0.78
Hearing impairment	0.092	0.33	0.09	0.34	−0.119	0.19	−0.119	0.12
Smoking	0.085	0.36	0.112	0.23	−0.120	0.21	0.035	0.68
Physical activity (*N*) ≥150 min/week of moderate-intensity aerobic activity or ≥75 min/week of vigorous-intensity aerobic activity	0.130	0.18	0.091	0.38	0.415	0.001	0.270	0.002
Traumatic brain injury	−0.148	0.12	−0.123	0.2	−0.105	0.25	0.028	0.74
Depression	−0.300	0.002	−0.295	0.004	0.029	0.77	−0.011	0.89

Age (years)			0.027	0.77			−0.432	0.002
Time from KT to assessment (days)/Duration of dialysis (months)			0.059 -	0.53 -			- −0.021	- 0.81
CCI			−0.219	0.16			−0.229	0.13
R^2^	0.227	0.005	0.25	0.38	0.273	0.02	0.500	0.001
R^2^ change	0.227	0.005	0.023	0.38	0.273	0.02	0.227	0.001

KT: kidney transplantation; PD: peritoneal dialysis; HD: hemodialysis; CCI: Charlson Comorbidity Index; *β*: Standardized Coefficients (Beta).

## Data Availability

The raw data supporting the conclusions of this article will be made available by the authors on request.

## References

[1] Kamide K. (2024). CKD could be a new risk factor of dementia. Hypertens. Res..

[2] Pépin M., Klimkowicz-Mrowiec A., Godefroy O., Delgado P., Carriazo S., Ferreira A.C., Golenia A., Malyszko J., Grodzicki T., Giannakou K. (2023). Cognitive disorders in patients with chronic kidney disease: Approaches to prevention and treatment. Eur. J. Neurol..

[3] Bugnicourt J.-M., Godefroy O., Chillon J.-M., Choukroun G., Massy Z.A. (2013). Cognitive disorders and dementia in CKD: The neglected kidney-brain axis. J. Am. Soc. Nephrol..

[4] Golenia A., Olejnik P., Żołek N., Wojtaszek E., Małyszko J. (2023). Cognitive Impairment and Anxiety Are Prevalent in Kidney Transplant Recipients. Kidney Blood Press. Res..

[5] Golenia A., Zolek N., Olejnik P., Wojtaszek E., Glogowski T., Malyszko J. (2023). Prevalence of Cognitive Impairment in Peritoneal Dialysis Patients and Associated Factors. Kidney Blood Press. Res..

[6] Golenia A., Żołek N., Olejnik P., Żebrowski P., Małyszko J. (2023). Patterns of Cognitive Impairment in Hemodialysis Patients and Related Factors including Depression and Anxiety. J. Clin. Med..

[7] Jurgensen A., Qannus A.A., Gupta A. (2020). Cognitive Function in Kidney Transplantation. Curr. Transplant. Rep..

[8] Rapa S.F., Di Iorio B.R., Campiglia P., Heidland A., Marzocco S. (2020). Inflammation and Oxidative Stress in Chronic Kidney Disease—Potential Therapeutic Role of Minerals, Vitamins and Plant-Derived Metabolites. Int. J. Mol. Sci..

[9] Tamura M.K., Tam K., Vittinghoff E., Raj D., Sozio S.M., Rosas S.E., Makos G., Lora C., He J., Go A.S. (2016). Inflammatory Markers and Risk for Cognitive Decline in Chronic Kidney Disease: The CRIC Study. Kidney Int. Rep..

[10] Zimmermann S., Mathew A., Bondareva O., Elwakiel A., Waldmann K., Jiang S., Rana R., Singh K., Kohli S., Shahzad K. (2024). Chronic kidney disease leads to microglial potassium efflux and inflammasome activation in the brain. Kidney Int..

[11] Rotondi S., Tartaglione L., Pasquali M., Ceravolo M.J., Mitterhofer A.P., Noce A., Tavilla M., Lai S., Tinti F., Muci M.L. (2023). Association between Cognitive Impairment and Malnutrition in Hemodialysis Patients: Two Sides of the Same Coin. Nutrients.

[12] Baumgart M., Snyder H.M., Carrillo M.C., Fazio S., Kim H., Johns H. (2015). Summary of the evidence on modifiable risk factors for cognitive decline and dementia: A population-based perspective. Alzheimer’s Dement..

[13] Litke R., Garcharna L.C., Jiwani S., Neugroschl J. (2021). Modifiable Risk Factors in Alzheimer Disease and Related Dementias: A Review. Clin. Ther..

[14] Livingston G., Huntley J., Sommerlad A., Ames D., Ballard C., Banerjee S., Brayne C., Burns A., Cohen-Mansfield J., Cooper C. (2020). Dementia prevention, intervention, and care: 2020 report of the Lancet Commission. Lancet.

[15] Ngandu T., Lehtisalo J., Solomon A., Levälahti E., Ahtiluoto S., Antikainen R., Bäckman L., Hänninen T., Jula A., Laatikainen T. (2015). A 2 year multidomain intervention of diet, exercise, cognitive training, and vascular risk monitoring versus control to prevent cognitive decline in at-risk elderly people (FINGER): A randomised controlled trial. Lancet.

[16] Rosenberg A., Mangialasche F., Ngandu T., Solomon A., Kivipelto M. (2020). Multidomain Interventions to Prevent Cognitive Impairment, Alzheimer’s Disease, and Dementia: From FINGER to World-Wide FINGERS. J. Prev. Alzheimer’s Dis..

[17] Bull F.C., Al-Ansari S.S., Biddle S., Borodulin K., Buman M.P., Cardon G., Carty C., Chaput J.-P., Chastin S., Chou R. (2020). World Health Organization 2020 guidelines on physical activity and sedentary behaviour. Br. J. Sports Med..

[18] Charlson M.E., Pompei P., Ales K.L., MacKenzie C.R. (1987). A new method of classifying prognostic comorbidity in longitudinal studies: Development and validation. J. Chronic Dis..

[19] Kaczmarek B., Ilkowska Z., Kropinska S., Tobis S., Krzyminska-Siemaszko R., Kaluzniak-Szymanowska A., Wieczorowska-Tobis K. (2022). Applying ACE-III, M-ACE and MMSE to Diagnostic Screening Assessment of Cognitive Functions within the Polish Population. Int. J. Environ. Res. Public Health.

[20] Lautenschlager N.T., Cox K.L., Flicker L., Foster J.K., Van Bockxmeer F.M., Xiao J., Greenop K.R., Almeida O.P. (2008). Effect of Physical Activity on Cognitive Function in Older Adults at Risk for Alzheimer Disease: A randomized trial. JAMA.

[21] Ratey J.J., Loehr J.E. (2011). The positive impact of physical activity on cognition during adulthood: A review of underlying mechanisms, evidence and recommendations. Prog. Neurobiol..

[22] Cai H., Li G., Hua S., Liu Y., Chen L. (2017). Effect of exercise on cognitive function in chronic disease patients: A meta-analysis and systematic review of randomized controlled trials. Clin. Interv. Aging.

[23] Chu N.M., Hong J., Harasemiw O., Chen X., Fowler K.J., Dasgupta I., Bohm C., Segev D.L., McAdams-DeMarco M.A., The Global Renal Exercise Network (2021). Chronic kidney disease, physical activity and cognitive function in older adults—Results from the National Health and Nutrition Examination Survey (2011–2014). Nephrol. Dial. Transplant..

[24] Bogataj Š., Mesarič K.K., Pajek M., Petrušič T., Pajek J. (2022). Physical exercise and cognitive training interventions to improve cognition in hemodialysis patients: A systematic review. Front. Public Health.

[25] Wallace L.M.K., Wallace L.M.K., Theou O., Theou O., Godin J., Godin J., Andrew M.K., Andrew M.K., A Bennett D., Bennett D.A. (2019). Investigation of frailty as a moderator of the relationship between neuropathology and dementia in Alzheimer’s disease: A cross-sectional analysis of data from the Rush Memory and Aging Project. Lancet Neurol..

[26] Manfredini F., Mallamaci F., D’arrigo G., Baggetta R., Bolignano D., Torino C., Lamberti N., Bertoli S., Ciurlino D., Rocca-Rey L. (2016). Exercise in Patients on Dialysis: A Multicenter, Randomized Clinical Trial. J. Am. Soc. Nephrol..

[27] Baggetta R., D’arrigo G., Torino C., ElHafeez S.A., Manfredini F., Mallamaci F., Zoccali C., Tripepi G., on behalf of the EXCITE Working group (2018). Effect of a home based, low intensity, physical exercise program in older adults dialysis patients: A secondary analysis of the EXCITE trial. BMC Geriatr..

[28] Belik F.S., Silva V.R.O.E., Braga G.P., Bazan R., Vogt B.P., Caramori J.C.T., Barretti P., Gonçalves R.d.S., Bôas P.J.F.V., Hueb J.C. (2018). Influence of Intradialytic Aerobic Training in Cerebral Blood Flow and Cognitive Function in Patients with Chronic Kidney Disease: A Pilot Randomized Controlled Trial. Nephron.

[29] McAdams-DeMarco M.A., Konel J., Warsame F., Ying H., Fernández M.G., Carlson M.C., Fine D.M., Appel L.J., Segev D.L. (2018). Intradialytic Cognitive and Exercise Training May Preserve Cognitive Function. Kidney Int. Rep..

[30] Chu N.M., Segev D., McAdams-DeMarco M.A. (2020). Interventions to Preserve Cognitive Functioning among Older Kidney Transplant Recipients. Curr. Transplant. Rep..

[31] Mortamais M., Portet F., Brickman A.M., Provenzano F.A., Muraskin J., Akbaraly T.N., Berr C., Touchon J., Bonafé A., le Bars E. (2013). Education Modulates the Impact of White Matter Lesions on the Risk of Mild Cognitive Impairment and Dementia. Am. J. Geriatr. Psychiatry.

[32] Pettigrew C., Soldan A. (2019). Defining Cognitive Reserve and Implications for Cognitive Aging. Curr. Neurol. Neurosci. Rep..

[33] Stern Y., Arenaza-Urquiljo E.M., Bartrés-Faz D., Belleville S., Cantillon M., Chetelat G., Ewers M., Franzmeier N., Kempermann G., Kremen W.S. (2020). Whitepaper: Defining and investigating cognitive reserve, brain reserve, and brain maintenance. Alzheimer’s Dement..

[34] Prakash J., Ryali V., Srivastava K., Bhat P., Shashikumar R. (2011). Cognitive reserve: The warehouse within. Ind. Psychiatry J..

[35] Håkansson K., Rovio S., Helkala E.-L., Vilska A.-R., Winblad B., Soininen H., Nissinen A., Mohammed A.H., Kivipelto M. (2009). Association between mid-life marital status and cognitive function in later life: Population based cohort study. BMJ.

[36] Kimmel P.L. (2001). Psychosocial factors in dialysis patients. Kidney Int..

[37] Agrawaal K.K., Chhetri P.K., Singh P.M., Manandhar D.N., Poudel P., Chhetri A. (2019). Prevalence of Depression in Patients with CKD 5 on Hemodialysis at A Tertiary Care Center in Nepal. J. Nepal Med. Assoc..

[38] Goh Z.S., Griva K. (2018). Anxiety and depression in patients with end-stage renal disease: Impact and management challenges—A narrative review. Int. J. Nephrol. Renov. Dis..

[39] Wong M., Kiss A., Herrmann N., Lanctôt K.L., Gallagher D. (2024). Modifiable Risk Factors Associated With Cognitive Decline in Late Life Depression: Findings From the Canadian Longitudinal Study on Aging: Facteurs de risque modifiables associés au déclin cognitif dans la dépression en fin de vie: Constatations de l’Étude longitudinale canadienne sur le vieillissement. Can. J. Psychiatry.

[40] Papakostas G.I. (2013). Cognitive Symptoms in Patients With Major Depressive Disorder and Their Implications for Clinical Practice. J. Clin. Psychiatry.

[41] You R., Ho Y.-S., Chang R.C.-C. (2022). The pathogenic effects of particulate matter on neurodegeneration: A review. J. Biomed. Sci..

[42] Peters R., Ee N., Peters J., Booth A., Mudway I., Anstey K.J. (2019). Air Pollution and Dementia: A Systematic Review. J. Alzheimer’s Dis..

[43] Chen H., Kwong J.C., Copes R., Hystad P., van Donkelaar A., Tu K., Brook J.R., Goldberg M.S., Martin R.V., Murray B.J. (2017). Exposure to ambient air pollution and the incidence of dementia: A population-based cohort study. Environ. Int..

